# The Danish Cardiovascular Screening Trial (DANCAVAS): study protocol for a randomized controlled trial

**DOI:** 10.1186/s13063-015-1082-6

**Published:** 2015-12-05

**Authors:** Axel Cosmus Pyndt Diederichsen, Lars Melholt Rasmussen, Rikke Søgaard, Jess Lambrechtsen, Flemming Hald Steffensen, Lars Frost, Kenneth Egstrup, Grazina Urbonaviciene, Martin Busk, Michael Hecht Olsen, Hans Mickley, Jesper Hallas, Jes Sanddal Lindholt

**Affiliations:** Elitary Research Centre of Individualized Medicine in Arterial Disease (CIMA), Department of Cardiology, University Hospital Odense, Odense, Denmark; Elitary Research Centre of Individualized Medicine in Arterial Disease (CIMA), Department of Clinical Biochemistry and Pharmacology, University Hospital Odense, Odense, Denmark; Department of Public Health and Department of Clinical Medicine, Aarhus University, Aarhus, Denmark; Department of Cardiology, University Hospital Odense, Svendborg, Denmark; Department of Cardiology, Vejle Hospital, Vejle, Denmark; Department of Cardiology, Diagnostic Centre, Regional Hospital Silkeborg, Silkeborg, Denmark; Elitary Research Centre of Individualized Medicine in Arterial Disease (CIMA), Department of Endocrinology, University Hospital Odense, Odense, Denmark; Department of Cardiology, University Hospital Odense, Odense, Denmark; Institute of Pharmacology, University of Southern Denmark, Odense, Denmark; Elitary Research Centre of Individualized Medicine in Arterial Disease (CIMA), Department of Cardiothoracic and Vascular Surgery, Odense University Hospital, Sdr. Boulevard 29, 5000 Odense C, Denmark

**Keywords:** Cardiovascular prevention, Screening, Aortic aneurysm, Coronary calcium score, Atrial fibrillation, Peripheral arterial disease, Benefit, Cost effectiveness, Public health

## Abstract

**Background:**

The significant increase in the average life expectancy has increased the societal challenge of managing serious age-related diseases, especially cancer and cardiovascular diseases. A routine check by a general practitioner is not sufficient to detect incipient cardiovascular disease.

**Design:**

Population-based randomized clinically controlled screening trial.

**Methods:**

*Participants*: 45,000 Danish men aged 65–74 years living on the Island of Funen, or in the surrounding communities of Vejle and Silkeborg. No exclusion criteria are used.

*Interventions:* One-third will be invited to cardiovascular seven-faceted screening examinations at one of four locations. The screening will include: (1) low-dose non-contrast CT scan to detect coronary artery calcification and aortic/iliac aneurysms, (2) brachial and ankle blood pressure index to detect peripheral arterial disease and hypertension, (3) a telemetric assessment of the heart rhythm, and (4) a measurement of the cholesterol and plasma glucose levels. Up-to-date cardiovascular preventive treatment is recommended in case of positive findings.

*Objective:* To investigate whether advanced cardiovascular screening will prevent death and cardiovascular events, and whether the possible health benefits are cost effective.

*Outcome:* Registry-based follow-up on all cause death (primary outcome), and costs after 3, 5 and 10 years (secondary outcome).

*Randomization:* Each of the 45,000 individuals is, by EPIDATA, given a random number from 1–100. Those numbered 67+ will be offered screening; the others will act as a control group.

*Blinding*: Only those randomized to the screening will be invited to the examination;the remaining participants will not.

*Numbers randomized:* A total of 45,000 men will be randomized 1:2.

*Recruitment*: Enrollment started October 2014.

*Outcome*: A 5 % reduction in overall mortality (HR = 0.95), with the risk for a type 1 error = 5 % and the risk for a type II error = 80 %, is expected. We expect a 2-year enrollment, a 10-year follow-up, and a median survival of 15 years among the controls. The attendance to screening is assumed to be 70 %.

**Discussion:**

The primary aim of this so far stand-alone population-based, randomized trial will be to evaluate the health benefits and costeffectiveness of using non-contrast full truncus computer tomography (CT) scans (to measure coronary artery calcification (CAC) and identify aortic/iliac aneurysms) and measurements of the ankle brachial blood pressure index (ABI) as part of a multifocal screening and intervention program for CVD in men aged 65–74.

Attendance rate and compliance to initiated preventive actions must be expected to become of major importance.

**Trial registration:**

Current Controlled Trials: ISRCTN12157806 (21 March 2015).

## Background

Although cardiovascular diseases (CVDs) have decreased during the last two decades, CVDs are still one of the most predominant causes of morbidity and mortality in the western world, including Denmark, where approximately 420,000 people have recognized symptoms [1, 2]. Due to an aging population, the decline in CVD incidence observed during the past decades has not led to a decrease in hospital admissions and health-related costs due to CVDs. The size of the Danish population is about 5.5 million, and approximately 14,000 people die annually from CVDs, compared to 16,000 deaths caused by cancer, the most common cause of death. The annual CVD-attributable costs of hospital admissions amount to DKK 4.6 billion, while pharmaceuticals amount to DKK 2.4 billion. In addition, an unknown number of visits to the general practitioner (GP) occur.

Screening for CVD has been discussed intensively [3, 4]. Ultrasound-based screening for abdominal aortic aneurysm (AAA) seems to reduce mortality [5], while evidence supporting screening for ischemic heart disease is lacking. Population-based screening of high-risk individuals with the intention to initiate preventive treatment has not been associated with a reduction in all-cause or cardiovascular mortality [6, 7].

To assess the risk and target preventive interventions, global risk scores, such as the HeartScore and the Framingham Risk Score, are recommended. These scores are useful for combining individual risk factors (age, gender, diabetes, smoking, blood pressure, and cholesterol levels) into a single quantitative risk estimate. In Europe, individuals with a 10-year risk of CVD death ≥5 % qualify for primary prevention, including lifestyle intervention and, in some cases, drug treatment. In individuals with a CVD death risk ≥10 %, drug treatment is more frequently recommended. However, in individuals older than 65 years, the risk scores should be interpreted more leniently due to a lack of evidence [8, 9]. Guideline-based recommendations on interventions for the elderly population are lacking despite the fact that more than 95 % of CVD deaths occur in people more than 65 years old. The European Guidelines on cardiovascular disease prevention 2012 acknowledge that “Prevention of CVD ideally starts during pregnancy and lasts until the end of life”, and states that “Studies have shown that preventive measures (i.e. BP lowering and smoking cessation) are beneficial up to advanced age.” However, elderly people have an increased risk of non-cardiovascular disease and all-cause death, and the guideline states “In persons older than 60, these thresholds should be interpreted more leniently, because their age-specific risk is normally around these levels, even when other cardiovascular risk factor levels are ‘normal’.”

Consequently, the primary aim of this so far stand-alone population-based, randomized trial will be to evaluate the health benefits and costeffectiveness of using non-contrast full truncus computer tomography (CT) scans (to measure coronary artery calcification (CAC) and identify aortic/iliac aneurysms) and measurements of the ankle brachial blood pressure index (ABI) as part of a multifocal screening and intervention program for CVD in men aged 65–74. Secondary aims will be the prospective observational studies of the prognostic importance of the calcification scores of the carotid, aorta, iliac, and femoral arteries, as well as the pathophysiological and translational biomarker studies that are made possible by biobanking.

### Calcified arteries: carotid, coronary, aortic, iliac, and femoral arteries

A low-dose CT scan without contrast can visualize calcifications of any artery. This technology was especially developed to evaluate for CAC, and several studies have demonstrated that the CAC scores improve the discrimination and reclassification of CAD above and beyond the traditional risk factors [10–14]. The prognostic value of grading calcifications of the carotid, aortic, iliac, and common femoral arteries is unknown. A few randomized clinical trials (RCTs) have evaluated the effect of statin treatment in asymptomatic patients with CAC, but the results are inconclusive [15, 16].

### Aneurysms

Level A evidence has shown that ultrasound-based screening for AAA in men aged 65–74 years in Denmark reduces AAA mortality by 66 % at a cost of DKK 1,500 to 5,500 per life-year gained [5, 17], and the procedure is being implemented in the UK, USA, and Sweden. By extending a non-contrast CT screening for CAC to include the abdominal aorta, the screening for AAA will incur a small additional cost, but it will also uncover thoracic aortic aneurysms (TAAs) and iliac aneurysms (IAs). Modern endovascular treatment may provide a low-risk intervention for these aneurysms. AAA, TAA, and IA will be diagnosed with close to 100 % sensitivity and specificity by the extended use of a non-contrast CT scan.

### Peripheral arterial disease (PAD)

Studies indicate that approximately 5–10 % of men above 60 years old show signs of PAD and the proportion increases with age. Although three of four patients are asymptomatic, approximately 25–30 % of the patients with PAD will die from CVD within a 5-year period regardless of whether they exhibit symptoms or not. An even higher proportion will need hospitalization due to CVD [18]. Efficient prevention of CVD-specific deaths, amputations, and other CVD events can be achieved through smoking cessation, exercise, a healthful diet, aspirin, lipid-lowering treatment, and treatment of elevated blood pressure [19, 20].

### Hypothesis

The primary hypothesis is that the offer of an extensive cardiovascular screening and intervention program fulfills the WHO criteria for screening [21], especially concerning the significance of the diseases, the treatment benefits, and the cost effectiveness from a national health care system perspective.

### Objectives

The primary objective of the study is to establish the health effect and the second is to evaluate the cost effectiveness of an advanced cardiovascular screening and intervention program for men aged 65–74 years in a randomized controlled trial.

## Methods/design

This is a population-based randomized clinically controlled screening trial, randomizing two thirds of the study participants to usual care without any screening (*control group*), while one third of the participants are invited to screening of CAC, aneurysms, PAD, atrial fibrillation, and traditional risk factors (*screening group*). The control group will not be informed about the trial.

### Power calculations

**Fig. 1:**
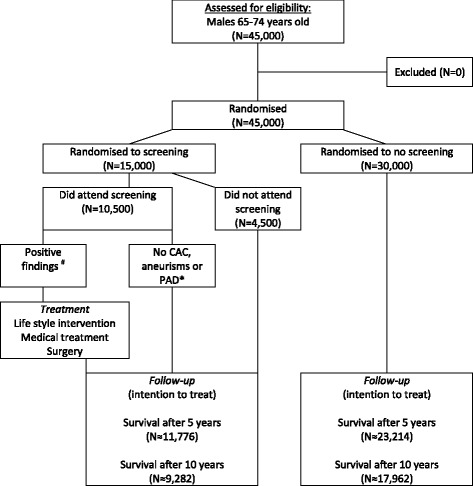
Expected flow chart of the men included in the DANCAVAS trial

A total of 45,000 (about 3x14,647) men are needed to detect a 5 % reduction in overall mortality (hazard ratio (HR) = 0.95) with the risk for a type 1 error = 5 % and the risk for a type II error = 80 %. We expect a 2-year enrollment, a 10-year follow-up, and a median survival of 15 years among the controls. The randomization ratio will be 1 invited:2 controls [22]. The attendance is assumed to be 70 % [5, 10] (Fig. 1).

### Randomization

Information on all people in the source population (civil registration number, name, and address) will be delivered by the civil personal registry (CPR). Randomization will be performed in EPIDATA by prospectively providing each individual a random number from 1–100. Those numbered 68–100 will be invited to participate in the screening program.

### Inclusion and exclusion criteria

The inclusion criteria are people of male gender, aged between 65 and 74 years, and who are living in the involved communities. There are no exclusion criteria.

### Screening

The central project secretariat will invite the selected individuals. A small questionnaire concerning life style, medical history, and the quality of life (QoL) a.o. will be enclosed in the invitation. Web-based booking is possible and recommended, but appointments can also be done by email or phone.

At attendance, informed consent will be obtained together with the questionnaire, weight, height, and waist circumference. HbA1c, lipid parameters, hemoglobin, creatinine kinase (CK), and alanine aminotransferase (ALAT) are measured and biobank blood samples are stored. Bilateral blood pressure will be recorded three times after 5 minutes of supine rest. The ankle blood pressure will be measured concurrently with the brachial blood. The CT scan will cover the area from the mandibular bone distally to the proximal third of the femur. Calcium scores for the coronaries will be calculated. The aorta will be visualized, and in the case of dilation, the maximal perpendicular outer to outer anteriorposterior (AP) and tranverse diameter will be measured. If no dilation is found, the AP diameter will be measured at five sites (ascending aorta, aortic arch, descending aorta, abdominal aorta just above the bifurcation, and iliac artery). The heart rhythm shown by the telemetry of the CT scanner is noted. Data is entered in a web database at the screening site.

**Fig. 2:**
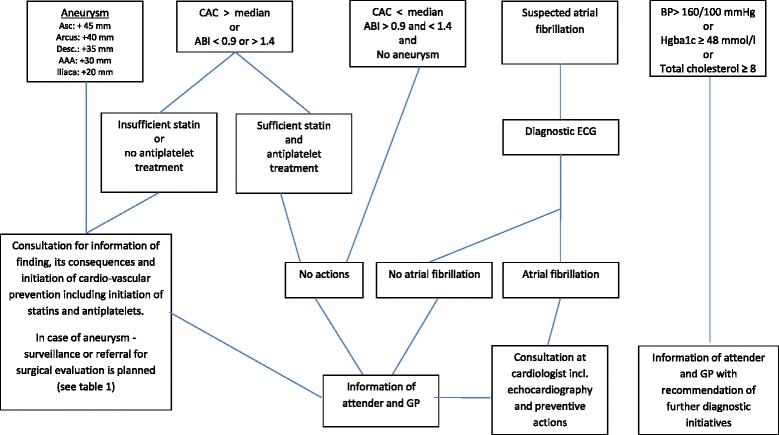
Algorithm after attending screening. Supplemental algorithm for aneurysms; see Table 1

Two days later the blood tests are loaded to the web-based database. A program evaluates all the individual findings, and sends lists to the secretary addressing which actions are to be taken (Fig. 2).

### Prophylactic actions after screening

For all screening locations, if the CAC is above the median or if ascending TAA, Arcus TAA, descending TAA, AAA, IA (definition: diameter ≥ 45, 40, 35, 30 and 20 mm, respectively) or PAD (Def.: ABI ≤ 0.9 or > 1.4) is detected, the patient will be informed at a follow-up visit of the finding and its potential implications. At this visit, the patient will be recommended suitable prophylactic measures, including smoking cessation, walking/exercise, a low-fat diet, and starting treatment with aspirin 75 mg/day (except in patients with anemia (hemoglobin below 6 mmol/L), patients with gastric ulcer within the prior three months, and patients with a daily use of non-steroidal anti-inflammatory drugs) and atorvastatin 40 mg/day (except in patients with elevated CK or ALAT, five and three times above normal reference level, respectively). If the ascending aorta, aortic arch, descending aorta, abdominal aorta, and iliac artery exceed a diameter of 65, 60, 55, 55, or 30 mm, respectively, the patient will be referred for a contrast-enhanced CT scan followed by a vascular surgical assessment at one of the four involved vascular centers that use these uniform size thresholds for the repair of asymptomatic aneurysms [23]. If an aneurysm is detected, but without an indication for surgery, biannual to every second year check-ups including a CT scan will be offered according to the algorithm shown in Table 1. Patients with prior unknown atrial fibrillation are prescribed anticoagulants and referred to echocardiography (Fig. 2).

**Table 1:** Algorithm after diagnosing an aneurysm in the DANCAVAS trial

Ascending aortic aneurysm:
+65 mm: Contrast-enhanced CT scan and referral for surgical evaluation
50–64 mm: Echocardiography for bicuspid aortic valve (BAV):
+ BAV: If so, contrast-enhanced CT scan and referral for surgical evaluation
- BAV: Annual non contrast-enhanced CT scan
45–49 mm: Non contrast-enhanced CT scan every 2 years
Aortic arch aneurysm
+60 mm: Contrast-enhanced CT scan and referral for surgical evaluation
55–59 mm: Biannual non contrast-enhanced CT scan
50–54 mm: Annual non contrast-enhanced CT scan
35–49 mm: Non contrast-enhanced CT scan every 2 years
Descending aortic aneurysm
+55 mm: Contrast-enhanced CT scan and referral for surgical evaluation
50–54 mm: Biannual non contrast-enhanced CT scan
45–49 mm: Annual non contrast-enhanced CT scan
35–44 mm: Non contrast-enhanced CT scan every 2 years
Abdominal aortic aneurysm
+55 mm: Contrast-enhanced CT scan and referral for surgical evaluation
50–54 mm: Biannual non contrast-enhanced CT scan
45–49 mm: Annual non contrast-enhanced CT scan
30–44 mm: Non contrast-enhanced CT scan every 2 years
Iliac aneurysm
Confirmation of diagnosis by vascular surgeon (JL)
+30 mm: Contrast-enhanced CT scan and referral for surgical evaluation
25–29 mm: Annual non contrast-enhanced CT scan

If no positive findings (CAC above the median, aneurysm, PAD, or atrial fibrillation) are detected, the participants will be informed of the findings by ordinary mail.

Independent of the above findings, the patients will be encouraged to contact their GP for further assessment if potential undiagnosed hypertension (systolic blood pressure ≥160 mmHg) [24], diabetes mellitus (HbA_1c_ ≥48 mmol/mol) [25], or significant isolated hypercholesterolemia (total cholesterol ≥8.0 mmol/L) are observed, as possible continuous medical treatments will be better managed by the GPs.

The GPs will be informed electronically of all negative and positive results and the initiated actions (Fig. 2).

### Annual follow-up

Annual follow-up will be conducted through data extraction from registries and the life style questionnaires that will be sent to the participants. Prescription records that show the implementation of pharmacological interventions, as well as hospitalization records and disease-specific mortality data, will be collected from the government maintained nationwide registries, as no delivery of prescribed medications, hospitalizations, and deaths is possible in Denmark without being recorded in these registries. These individual-based data are available for researchers. Consequently, these registries have been continuously subjected to an in-depth validation process and have been proven valid regarding data on CVD and mortality [26–32].

Patients with aneurysms that do not exceed the size threshold for a vascular surgical evaluation referral will be offered a biannual to every second year CT (Table 1). The attendance rates for these control scans are documented in the RCTs performed previously in Denmark to be 95 %.

### Pilot studies

Supervised measurements of the CAC scoring, the diameters of thoracoabdominal aorta and iliac arteries, and the ABI will be performed by senior examiners in the first 50 cases, followed by interobserver validation in the next 50 cases. If the mean differences in the CAC score, aortic diameter, and ABI are below 10 %, 2 mm, and 15 %, respectively, unsupervised examination will be allowed; otherwise, the training and validation procedure will be repeated. After the first 500 examinations, re-validation of the interobserver agreement will be repeated.

The age-specific median for the CAC score will be established after the first 500 examinations. Non-contrast CT and ultrasound will be performed in the first 500 men, and using ultrasound as the gold standard, the concordance between non-contrast CT and ultrasound in the detection of AAA will be analyzed.

The screening method for hypertension (after 5 minutes of supine rest) will be validated in the first 500 men with blood pressure measurements of patients in a seated position in an isolated room as the gold standard. In both cases, the blood pressure will be measured three times. In addition, 24 hour blood pressure measurements will be taken in a subgroup.

Personal contact information, the randomization process, the central secretary administration, staff education, the acceptance rates to the screening invitation, the standardization of screening and treatment protocols, the systems for collecting initial and follow-up data, the response rates to the questionnaires, quality control of the procedures, and the organization of trial data will be tested and evaluated in the first 2,000 male residents of Odense recruited into the study.

### Outcome variables and statistical analyses

The entire study population, the controls as well as the screening group, will be monitored for a period of 10 years. The primary outcome variable is overall mortality, while hospitalizations and deaths from cardiovascular diseases (cerebrovascular, cardiac, aneurysm, or other vascular) are the secondary endpoints. Specific causes of deaths and hospitalizations will be analyzed respectively, and not as composite endpoints.

The endpoints are compared for the two groups using a Cox proportional hazards regression analysis by the “intention-to-treat” principle.

Cancer incidence and cancer-related deaths will be compared between the two groups as a safety outcome as a possible consequence of mass CT scanning.

An independent endpoint committee will review registry data on the causes of death and data from the Danish National Patient Register concerning hospital admissions; supplemental data will be requested from hospitals and the GP if needed.

### Cost effectiveness evaluation

Analysis will be based on national registries of health care utilization and the outcomes of the clinical analysis and undertaken from a health care sector perspective after 5 and 10 years of follow-up, respectively. The cost effectiveness analysis will be based on all-cause mortality as the primary outcome and disease-specific mortality as a secondary outcome [33]. The cost-utility analysis will be based on quality adjusted life years using Danish preference weights of the normal population [34]. In addition, the lifetime perspective will be analyzed in a separate decision analytic model [35]. Finally, pilot studies involving 1,000 randomly selected women aged 65–74 years and 1,000 men aged 60–64 will be performed for health economic cost effectiveness modeling, in order to evaluate the consequences of choosing these alternative target groups.

### Ethical considerations

The medical examination will not cause any notable inconveniences or any definite risk. However, several large epidemiological studies do suggest that radiation exposure is associated with a slightly increased risk of cancer. The best-studied cohort is the Japanese atomic bomb survivor cohort. In a group exposed to radiation doses of 5 – 100 mSv (a mean dose of 29 mSv), 4,406 solid cancers were observed between 1958 and 1998, an excess of 81 solid cancers over the expected cancer rates. This finding corresponds to an excess relative risk of 2 % [36]. No large studies involving medically exposed adult cohorts are available, but a linear no-threshold model has been considered. Thus, there may be no minimal radiation dose for an increased cancer risk, and the risk increases linearly with the radiation dose. The average dose in our pilot study was 1 mSv [10]. For comparison, a typical dose of a mammogram is 0.2 mSv, the annual background radiation dose in Denmark is 3 mSv, and the average annual limit for radiation workers is 20 mSv [37]. According to the Danish National Committee on Biomedical Research Ethics, a radiation dose of 0.1–1 mSv to subjects under 50 year of age is associated with an overall cancer risk in the magnitude of 1 in 100,000, and to subjects older than 50 years, the radiation dose can be increased by a factor of 5 to 10.

An offer for screening is known to reduce the quality of life in the period leading up to the examination, but the effect fades out in the absence of a positive finding. It is unclear if a sustained reduction in the quality of life is caused by the diagnosis itself or by comorbidity, but the reduction is modest. Such “secondary side effects” should be weighed against the prophylactic benefits that might be achieved. A more serious problem in connection with the detection of aneurysms is the risk of death caused by rupture of the aneurysm in conservatively treated cases and perioperative deaths in cases where the AAA might not have otherwise ruptured. This serious ethical problem has no solution at present. We hope that the studies with the planned biobank will facilitate the development of a prognostic model.

Participants are mature men who are fully capable of deciding whether to accept or reject the invitation, and efforts have been made to provide a comprehensive explanation of the study in the invitation.

Participants will be given time to consider their participation before deciding and are informed that they may bring a companion, preferably a spouse/partner. In case of a positive finding, all participants will be offered an in-depth outpatient interview as soon as possible. At the consultation, information will be given concerning the prognosis of the positive finding and the need and benefit of prophylactic measures. Statin treatment may increase the risk of diabetes mellitus by 9 % (from 1.12 % to 1.22 % per year, adapted from [38]). However, this risk is outperformed by the decreased risk of development of ischemic heart disease and stroke by 30 % and 19 %, respectively (from 1.29 % to 1.00 % per year, and 0.55 % to 0.45 %, respectively, adapted from [6]). Aspirin increases the risk of hemorrhage (from 0.07 % to 0.10 % per year) while decreasing the risk of CVD (from 0.57 % to 0.51 % per year) [39]. The side effects should be weighed against the risk reduction achieved. A specific dilemma is the situations in which AAA is diagnosed and requires surgery, which carries a 1–3 % mortality risk, which should be weighed against the mortality risk of approximately 90 % in AAA ruptures. When there is an AAA surgery indication, the patient is informed of the surgical risks and the risks of conservative treatment, and a surgery date is set. One of the project managers has experience in performing this function.

As a part of the study, a biobank will be organized. This will be maintained for a 15-year period, and data will subsequently be anonymous.

The protocol was approved by the Regional Scientific Ethical Committee for Southern Denmark (S-20140028) and the Data Protection Agency. It is conducted in accordance with the Declaration of Helsinki. Written informed consent was obtained from each participant.

## Discussion

The target group was determined by cynical considerations of where the benefit was most likely to be highest and most cost effective. Younger men were excluded, as 85 % of all cardiovascular deaths in Denmark occur after the age of 65, the CAC scores, prevalences of aneurysms and PAD increase rapidly after the age of 65, and screening for AAA has proven beneficial and cost effective in men aged 65–74.

Women were excluded, as although they increasingly die of CVD, it happens in general much later in life. Thus CVD-related morbidity and mortality in this age group is much higher in men — at least in Denmark — and screening for AAA is cost effective in men in this age group, while the prevalence in Danish women is 1–2 out of 1,000 and thus most likely to be cost ineffective. However, the decision was difficult, and random samples of women aged 65–74 and men aged 60–64 will be invited in order to be able to estimate the benefit and cost-effectiveness by modeling*.*

The trial compares active systematic preventive action based upon finding of subclinical arterial lesions in the coronary and lower limb arteries and the aortoiliacal vessels as well as classic risk factor management. If some are started by GPs without DANCAVAS criteria fulfilled, we consider that to be similar to what is happening in the control group. Consequently, it seems difficult to identify others in high risk without expensive angiographic examinations. Consequently, it could be questioned whether if no lesions were diagnosed, that existing preventive medication could be stopped. However, we decided on a pragmatic approach, as the patient could be worried if preventive actions were taken away, and his GP could become hostile for DANCAVAS recommendation concerning other patients of the GP.

We decided to define hypercholesterolemia as > 8 mmol/L, which is relatively high. However, if any lesion was detected, statins were recommended regardless of plasma cholesterol. If no lesion is observed when a patient has very high cholesterol at the age of 70, it could be debated whether they should be treated at all, so we chose this high level.

### Financial and budgetary administration

The participating sites will fund the CT scans and room facilities, while the study organization will cover the costs for the study staff, hemoglobin, HbA_1c_, lipid, CK, and ALAT measurements, as well as biobanking. The obtained funding will be administered by OUH, where the project secretary will be located. Expenses are primarily paid for by funds from public and private donations and sponsorships. The project managers have no economic affiliation to any of the foundations. Participants will not receive any remuneration or reimbursements for transportation costs.

### Expected number of examinations, operations, and visits

Four screening sites are established in Svendborg, Odense, Vejle, and Silkeborg. At each site, 37 men will be invited at 10-minute intervals on each of the days scheduled for screening with a total of 284 screening days. If each site can provide two weekly screening days, enrollment may be completed within one year. Individuals with positive findings will attend a 20-minute outpatient appointment for information and initiation of preventive actions instruction. Two days a week is assumed needed for these outpatient appointments.

One in every 10 cases of AAA is expected to need referral for surgical assessment; among these referred cases, 90 % are expected to undergo surgery. These surgical cases are estimated to add 1–2 AAA operations to the surgery schedule per month per department. The vascular surgery departments do not anticipate any problems with the handling of these cases due to the prevention of emergency cases.

### Organization

*The Executive Committee*, consisting of the cardiologist Axel Diederichsen, biochemist Lars M. Rasmussen, and the vascular surgeon Jes S. Lindholt, will handle the decisions regarding the administration, budget, overall organization, data, use of biobank, and principles for authorships.

*The Steering Committee* will consist of the members of the executive committee, Michael Hecht Olsen as the expert in cardiovascular risk factors, Hans Mickley as an expert in ischemic heart disease, Jesper Hallas as the pharmaco-epidemiologist, a senior statistician who will be collaborating with Jesper Hallas, the health economist Rikke Søgaard, and one representative from each screening site. All practical issues concerning the screening, follow-up, biobank, and data sampling will be handled by the steering committee. In addition, the steering committee will participate as authors in the reporting of the primary endpoints.

*The Advisory Board* consists of the following international experts who have various special interests in specific areas: cardiologist Peter Libby (Brigham and Women’s Hospital, Boston, MA, USA), cardiologist Raimund Erbel (West-German Heart Center Essen, Germany), statistician Simon Thompson (MRC statistical unit, Cambridge, UK), health economist Dorthe Gyrd-Hansen (University of Southern Denmark), translational CV researcher Jean B Michel (Hôpital Xavier Bichat, Paris, France), and translational AA researcher Guo Ping Shi (Brigham and Women’s Hospital, Boston, USA).

### Data registration

Data registration will be electronic and based on numeric codes established by the project secretary and kept in a locked room at CIMA. The database will be stored on an internal hospital drive to avoid the risk of data loss in case of a technical failure. Only the project secretary and the independent data review committee members will have access to the complete database. However, the executive and steering committees will have access to data concerning the invited group to secure follow-up and allow for observational studies.

### Strengths and limitations

There are no exclusion criteria in the study, and this implies that patients with known CVD, such as former stroke and myocardial infarction, may also be invited to participate. This might be superfluous, because these individuals have documented CVD, and preventive care should have already been established. However, these patients do have an increased prevalence of aneurysms and may therefore benefit from the screening [40–42]. This possible benefit will be explored in detail in subgroup analyses. Another limitation is the lack of a non-imaging experimental group. Thus, in case there are benefits to the screening, we will be unable to differentiate whether the benefit was due to the imaging findings or due to treatment of classical risk factors, such as hypertension. However, in many of these elderly subjects, primary medical intervention is controversial, and the decision is left to the individual subjects with the GP minimizing the difference from the usual care. Additionally, in this study of males aged 65–74 years, the major problem of unexpected sudden deaths among young men is not addressed. However, as the population gets older, this study will provide important knowledge about initiating prevention among retired men.

### Feasibility

The two executive committee members, Axel Diederichsen and Jes Lindholt, have previously organized and implemented similar population studies concerning CAC, AAA, and PAD [10, 43, 44].

In addition, several experts assist the members of the executive committee: Michael Hecht Olsen has experience investigating risk factors in large clinical trials and population-based studies. Hans Mickley has important scientific knowledge in ischemic heart disease. Rikke Søgaard has experience in evaluating and modeling the cost effectiveness of screening programs. Jesper Hallas is experienced in exploiting the unique Danish pharmaco-epidemiological possibilities, while local experts are securing practical feasibility of the project at the specific screening sites. Lars Melholt Rasmussen is an expert in biochemistry and biobanking and, as the head of the Elitary Research Centre CIMA, he will perform the independent administration of this complex project.

Meetings with the advisory board, which consists of a multidisciplinary team of internationally recognized researchers, covering cardiovascular screening, health economics, advanced statistics, and translational cardiovascular medicine will design and plan the optimal method of data sampling including biosamples for scientific investigation. The design and plan will be implemented by the scientific committee and, finally, managed, analyzed, and reported by the specific writing committees organized by the executive committee.

## Trial status

In October 2014, the pilot study started at the site in Odense University Hospital to test the screening methods and logistics. At New Year 2014/2015, the program was running smoothly. However, every sixth appointment needs to be left unbooked in order to minimize severe delays. By May 2015, 1,200 participants had been included.

Two afternoon/evenings weekly are used for the screening. The participants visit four different rooms during the session. In the first room general information about the study is given, and informed consent together with a short questionnaire interview is obtained. In the second room measurements of brachial and ankle blood pressure as well as abdominal ultrasound scanning for AAA for validation through the pilot study are scheduled. In the third room the CT scans and measurements of CAC score and aortic diameters are performed. The heart rhythm is recorded from the telemetry of the CT scanner and subsequently noted. In the fourth and last room the required blood samples are drawn.

All data are collected in the web database, and a program performs an automatic interpretation. In patients with minor “positive” findings (CAC above the median, small aneurysm or PAD) and inadequate preventive medical treatment, a consultation with a nurse is arranged. During this consultation, the patients are advised to adopt a healthful lifestyle and medication is prescribed. Patients with major “positive” findings (like large aneurysms or atrial fibrillation) are referred for further diagnostic examinations (contrast CT scan and/or echocardiography) and a consultation with a physician and surgery if needed.

The remaining patients are informed of the “negative” findings in writing and encouraged to see their GP if potential undiagnosed hypertension, diabetes mellitus, or hypercholesterolemia are suspected. The GPs will be informed electronically of all negative and positive results and the initiated actions.

The second screening site is expected to start in June 2015 and the third in September 2015, while the start-up time of the fourth site is still unknown.

## Abbreviations

AAA: abdominal aortic aneurysm
ABI: ankle brachial blood pressure index
CIMA: Elitary Research Centers of Individualized Medicine in Arterial Disease
CVD: cardiovascular disease
CT: computer tomography
DKK: Danish Crowns
HR: hazard ratio
HbA1c: glycated hemoglobin
IA: iliac aneurysm
CAC: coronary artery calcification
PAD: peripheral arterial disease
QoL: quality of life
mSv: millisievert
TAA: thoracic aortic aneurysm
